# A Case Report of Rapidly Necrotizing Fasciitis Post-Falling Down Treated Reconstructively

**DOI:** 10.7759/cureus.28055

**Published:** 2022-08-16

**Authors:** Nancy Zeaiter, Deoda Maassarani, George Ghanime, Ziad Sleiman

**Affiliations:** 1 Plastic and Reconstructive Surgery, Lebanese University, Beirut, LBN; 2 Plastic Surgery, Lebanese Hospital Geitawi UMC, Aschrafieh, LBN; 3 Plastic and Reconstructive Surgery, Lebanese Hospital Geitawi UMC, Achrafieh, LBN

**Keywords:** case report, fasciotomy, surgical debridement, group a streptococcus, necrotizing fasciitis

## Abstract

Necrotizing fasciitis (NF) is a necrotizing soft tissue infection that can result in fast tissue loss, necrosis, and potentially fatal acute sepsis. Diabetes, cancer, alcohol abuse, and chronic liver and renal disease are all risk factors for NF. In this case report, a 19-year-old man with a negative past medical and surgical history was diagnosed with aggressive rapidly progressive necrotizing fasciitis of the left lower extremity after a recent history of falling down from a skateboard. A successful treatment with long-term debridement surgeries followed by reconstructive surgery with skin grafting was made. Although the severity of this condition, the patient was able to resume a normal range of motion of the concerned extremity. NF has been described in the literature, but early diagnosis, which is crucial for successful management, rests a challenge.

## Introduction

Hippocrates originally characterized necrotizing fasciitis (NF) in the fifth century, and it is a life-threatening soft tissue illness. The etiology has been known for ages, and Joseph Jones, a former Confederate Army surgeon, coined the term "Necrotizing Fasciitis" in 1871 [1].

NF is a rare bacterial inflammation (infection) that can destroy the skin and underlying tissues (connective tissue, subcutaneous fat, muscles, and muscle membranes). The disease can be very dramatic, with shock and damage to internal organs. Left untreated, the disease can lead to death. Usually, necrotizing fasciitis develops very quickly and can become life-threatening within hours or days. Immediate treatment and hospitalization are urgent, and usually, the inflamed tissue must be surgically removed [2].

There are two types of NF. Type I is polymicrobial, whereas type II is monomicrobial. Group A streptococcus (type 2 NF) is the most common cause of NF, and it can lead to streptococcal toxic shock syndrome (STSS), which is characterized by shock and multiple organ failures caused by a toxin produced by group A streptococcus. NF and STSS are occasionally seen together [in 40% of NF patients and 6% of other individuals (p0.001)] [3]. Differentiating NF from other soft tissue infections is notoriously tough, but it is critical since NF is a medical emergency that requires immediate and intensive surgical debridement. As a result, this condition puts physicians' diagnostic abilities and surgical tenacity to the test.

Group A streptococci live on the skin and throat of many people without causing harm, but can sometimes cause mild and, exceptionally, severe infections. Necrotizing fasciitis is one of the most serious infections caused by group A streptococci because the bacterium creates large amounts of toxins in the body. A combination of different bacteria is often the cause. Inflammation can spread from superficial as well as internal tissue damage. The pathogens can get into the soft tissue through small cuts, scratches, other wounds, or burns [4]. The bacteria can also come with the blood from other parts of the body and colonize the affected area. It is often not possible to find out how the bacterium got into the body. As a rule, the body's immune system succeeds in killing bacteria that enter the body. That is why it is often older people or people with a weakened immune system who are affected by the disease. However, necrotizing fasciitis can also occur in young, healthy people [5].

Symptoms of necrotizing fasciitis can appear very quickly - within a day - after a cut or other wound in the skin. The first and typical symptom of the disease is the rapid onset of severe pain in the infected area. The affected patients develop fever and chills, and the painful area may be red, slightly swollen, warm, and with overlying blisters. As the necrotizing fasciitis progresses, the inflamed area may turn black and blue, and it can be accompanied by shock due to low blood pressure. This leads to impaired consciousness, confusion, difficulty concentrating, cold sweats, and dizziness, or streptococcal toxic shock syndrome (STSS) [6]. If patients are not treated quickly enough, life-threatening internal organ damage can develop.

## Case presentation

A previously healthy 19-year-old Caucasian male was transferred to Lebanese Hospital Geitaoui for consultation and further reconstructive interventions, after being diagnosed with rapidly progressive necrotizing fasciitis of the left lower extremity in another local hospital. 

Prior to the transfer

**Figure 1:**
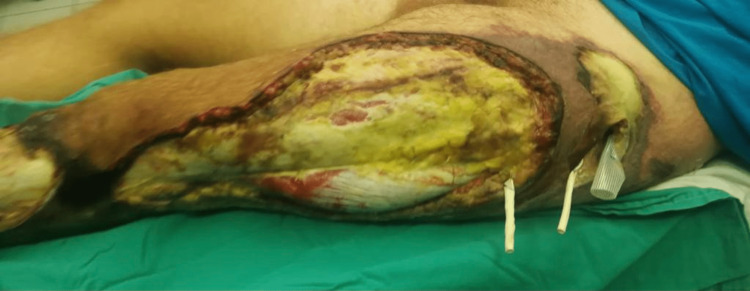
Clinical aspect of the concerned extremity immediately after fasciotomy (five days from the accident).

**Figure 2:**
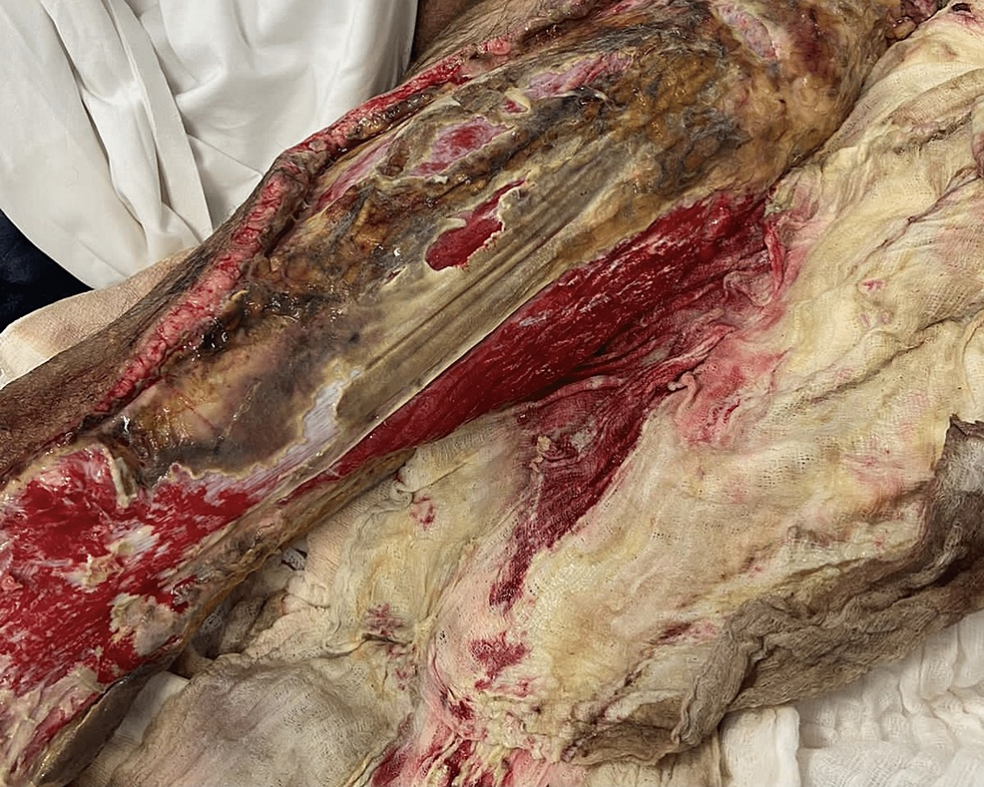
Clinical aspect of the concerned extremity immediately after debridement.

The patient had a recent history of falling down from a skateboard, with resultant abrasions with an erythematous violaceous wound with blisters developed on his left lower extremity within 24 hours of the accident. After a few days, he presented to the hospital with fever, hypotension (systolic blood pressure: 50 mmHg), tachycardia (heart rate: 110 beats/minute), tachypnea, and desaturation. On physical exam, the patient’s left lower extremity was swollen and indurated, along with patches of skin necrosis and crepitus. Significant abnormal lab results were documented (Table 1). Immediately after the presentation, resuscitation was done, and the patient was admitted to the intensive care unit, as a case of septic shock resulting from rapidly progressive necrotizing fasciitis of the left lower extremity. Broad-spectrum antibiotics (Meropenem and Vancomycin) were started following the administration of a single dose of Amikacin and Ceftriaxone. Decompression fasciotomy of leg compartments was done one day after admission, followed by surgical debridement the day after. Blood cultures were negative, and tissue cultures showed Streptococcus Pyogenes. Antimicrobial de-escalation was performed accordingly: the patient was switched to Tigecycline. The clinical result after fasciotomy and debridement is shown in Figures 1, 2 respectively. Hence, the patient became hemodynamically stable with resolution of the acute kidney injury (creatinine: 0.7 mg/dl) and improvement of inflammatory markers (WBC: 10,000/microliter).

**Table 1:** Significant abnormal lab results.

Lab test	Result
White blood cells (WBC)	18,000 [WBCs per microliter]
Neutrophils	73%
Hemoglobin	12.9 [g/dL]
Sodium	132 [mEq/L]
Creatine phosphokinase test (CPK)	1347 [U/L]
C-reactive protein (CRP)	15 [mg/dL]
Creatinine	3.87 [mg/dL]

After transfer to Lebanese Hospital Geitaoui

**Figure 3:**
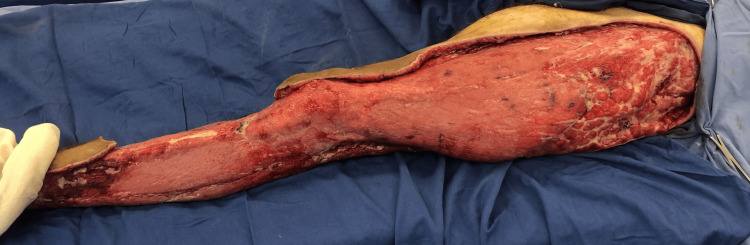
Clinical aspect of the concerned extremity after the last session of debridement.

On admission to the hospital, two weeks after the accident, the patient was hemodynamically stable and with normal labs. Empiric antibiotics, Piperacillin/ Tazobactam (Zocyn®, Taiho Pharmaceuticals, Japan), and Tigecycline (Tygacil®) were administered. The patient was maintained on a high protein and a high-caloric diet. Serial debridements (a total of three sessions), after obtaining written informed consent, were made for one week. Between one session and another, a 24-hour break took place. Each session took approximately two hours. Adequate removal of all necrotic and nonviable tissue was done. No progression of necrosis was shown after the third session. Tissue cultures showed the presence of Morganella morganii and Enterobacter cloacae; therefore, antibiotic administration was adjusted accordingly: Vancomycin was added. Dressings with silver sulfadiazine (Flamazine®, Smith & Nephew Pharmaceuticals Ltd. Origin, UK) and Jelonet paraffin gauze (Smith & Nephew Pharmaceuticals Ltd. Origin, UK) were done three times per week. The results after adequate debridement were satisfactory (Figure 3). After two weeks after the final debridement, there was a good formation of granulation tissue. Moreover, tissue cultures became negative. At this point, three sessions (48-hour break between each session) of split-thickness skin grafts were done to cover the large defect. The aim of dividing the grafting procedure into multiple sessions was to limit blood loss from the donor site. The grafts were removed from the right thigh by a dermatome. The grafts removed were of medium thickness (0.013 inches). A thick (0.019 inch) graft was also taken to cover the knee joint. Dressings on the skin grafts were done with Betadine cream and Jelonet.

**Figure 4:**
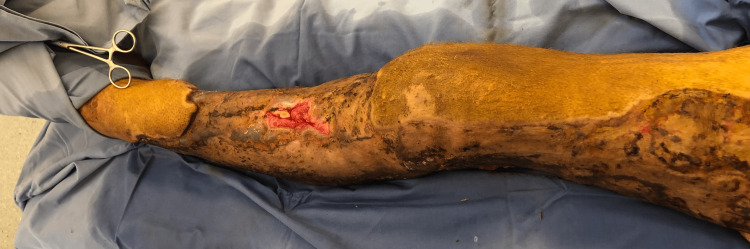
Clinical aspect of the concerned extremity after two weeks of skin grafting.

Two weeks after, complete healing of the skin grafts ensued (Figure 4) and the patient was discharged home after two months of hospitalization. Physiotherapy sessions were advocated and compression garments with silicone were prescribed to reduce scarring. Six months later, a final follow-up was made and the patient had a full range of motion in his concerned extremity with grade four power of muscle strength. The patient was satisfied and able to completely resume a normal quality of life. 

## Discussion

More than 500 to 1,000 instances of NF are identified each year in the United States, according to the US Centers for Disease Control and Prevention (CDC) [3]. However, because there are so many synonyms for these items, it is impossible to know how accurate this estimate is. According to reports, the yearly rate of NF is 0.40 cases per 100,000 people [7]. This rate has recently been rising at an exponential rate [8]. The infection causes NF, and predisposing factors include medications, hypersensitivity, vascular issues, burns, insect bites, needle stick injuries, and trauma [9]. In individuals with immunosuppression, diabetes, cancer, drug addiction, or chronic renal disease, NF can cause severe sepsis [10]. According to several studies, intravenous drug use is a major risk factor for NF [11]. NF is more prevalent in men in the winter, despite the fact that cases of NF caused by Vibrio vulnificus are more common in the summer [12]. It may affect anybody at any age; however, the risk increases as one become older. About half of the patients have had a skin abrasion, a quarter has had physical trauma, and seventy percent have one or more chronic conditions. A single lower limb accounts for half of the instances, whereas a single upper limb accounts for one-third [8]. 

Tenderness, edema, erythema, and discomfort at the afflicted location, which mirrors non-severe soft tissue infections (NSTIs) such as cellulitis and erysipelas, make NF difficult to identify in the early stages [9]. The cardinal NF symptom is intense pain at the outset that is out of proportion to physical findings [8,12,13]. Vibrio and Aeromonas are well-known aquatic pathogens that induce NF in people with chronic diseases, particularly in the liver, with a high death rate. The key to narrowing down the possible organism is getting a thorough history of saltwater exposure or fish stings in patients with liver or spleen disease [14].

Fever (>38°C) is seldom present (44%), although tachycardia (>100 beats/min) is common (59%), and hypotension (<100 mm Hg) (21%) and tachypnea (>20/min) (26%) are occasionally present. These three aberrant vital signs point to NF rather than NSTI [15]. Although NF can affect any part of the body, it is most frequent in the extremities (36-55%), trunk (18-64%), and perineum (up to 36%) [16]. Erythema (80%), induration (66%), soreness (54%), fluctuation (35%), skin necrosis (23%), and bullae (11%) are all seen in infected areas [15]. When comparing NF to NSTI, the positive probability ratio for the presence of bullae is 3.5. In another study [17], NF patients had more tense edema (23% vs 3%, p=0.0002), purple skin coloring (10% vs 1%, p=0.02), and sensory or motor loss (13% vs 3%, p=0.03) than NSTI patients. Skin necrosis was found in 6% of NF patients compared to 2% of NSTI patients. The first physical signs of NF are often erythematous and ecchymotic skin lesions, but these can quickly progress to hemorrhagic bullae, which signal the obstruction of deep blood vessels in the fascia or muscle compartments; consequently, the presence of bullae is a critical diagnostic clue. Ludwig's angina (submandibular space) and Fournier's gangrene (scrotum and penis or vulva) are examples of NF variations that affect particular parts of the body and can have a rapid start and severe clinical course. There are currently no laboratory parameters specific to NF. A so-called Laboratory Risk Indicator for Necrotizing Fasciitis (LRINEC) score has been proposed to classify the average risk of NF [18]. Patients with an LRINEC score of six or above need to have a detailed examination to rule out necrotizing fasciitis [19]. In our case described above, the LRINEC score at admission was 10 (CRP 15 mg/L, white cell count 18,000 per microliter, hemoglobin 12.9 g/dL, sodium 132 mEq/L, creatinine 3.87 mg/dL, glucose 90 mg/dL) which is highly indicative of NF.

When compared to situations in which surgery is postponed for even a few hours, surgical debridement is the cornerstone of NF therapy and results in much lower mortality [16]. Patients with NF should be sent to the operating theatre as soon as feasible for a "search and destroy" mission of vigorous and comprehensive debridement. Tissues that have been infected should be carefully resected until there is no more sign of infection. The most essential factor in determining survival is the initial operation, and the wound must be thoroughly examined following the initial debridement. If further debridement is required, the patient must be taken back to the operating room as soon as possible. After the first debridement, a "second-look" procedure is usually performed 12 to 24 hours later. Patients with NF may need anything from five to forty surgeries, according to one research, with an average of 33 debridements and grafting procedures required [7]. It is preferable to remove the tissues with acceptable margins rather than leaving just actively infected or necrotic tissue since this will reduce the risk of recurrence from the remaining infected tissue. After debridement, skin grafts are applied as a second-stage technique once a clean, granulating recipient bed has developed [20]. In light of our experience, it is recommended that the idea of radical debridement with immediate skin grafting may be usefully implemented in the treatment of necrotizing fasciitis.

## Conclusions

NF is a rare bacterial inflammation (infection) that can destroy the skin and underlying tissues (connective tissue, subcutaneous fat, muscles, and muscle membranes). The disease can be very dramatic, with shock and damage to internal organs. In this case report, serial debridement with adequate antibiotherapy followed by reconstructive surgeries were made. An early diagnosis remains challenging and crucial for salvaging the affected area.
